# Thoracic Endovascular Aortic Repair for Aortoesophageal Fistula after Covered Rupture of Aortic Homograft: A Durable Option?

**DOI:** 10.12945/j.aorta.2017.16.044

**Published:** 2018-09-24

**Authors:** Michal Nozdrzykowski, Jens Garbade, Steffen Leinung, Andrej Schmidt, Friedrich-Wilhelm Mohr, Michael A. Borger

**Affiliations:** 1 Department of Cardiac Surgery, Heart Center, University of Leipzig, Leipzig, Germany; 2 Department of Visceral and Thoracic Surgery, Park Hospital, Leipzig, Germany; 3 Division of Interventional Angiology, University Hospital, Leipzig, Germany

**Keywords:** TEVAR, Aortoesophageal fistula, Graft infection

## Abstract

A 63-year-old woman underwent replacement of the aortic root, ascending aorta, and partial arch due to Type A aortic dissection. Shortly thereafter, a replacement of the distal aortic arch and descending aorta was performed. Three years later, the patient developed an aortoesophageal fistula (AEF) resulting in re-replacement of the distal aortic arch and proximal descending aorta with a cryopreserved aortic homograft. Six weeks post-discharge, the patient was readmitted due to recurrent AEF. A thoracic endovascular stent graft was implanted to cover the aortic rupture, followed by correction of an esophageal lesion. The patient was monitored closely over time.

## Introduction

Although it is a rare clinical condition, aortoesophageal fistula (AEF) presents problems to therapy because of the high rates of morbidity and mortality associated with surgical management. Therefore, less invasive approaches that reduce perioperative mortality have been evaluated, with special attention given to thoracic endovascular aortic repair (TEVAR). However, this technique has important limitations in treating AEF, mainly due to a high risk of graft contamination. Here, we describe a case of recurrent AEF treated with TEVAR stenting of a cryopreserved aortic homograft replacement of the aortic arch and Hemashield prosthesis replacement of the descending aorta.

## Case Presentation

A 63-year-old woman underwent emergency replacement of the aortic root with a biological valve conduit (Medtronic Freestyle-Aortic-Root Modell 995, Gr. 23 mm, Medtronic Inc., Minneapolis, MN) as well as ascending aorta and partial arch replacement (Hemashield prosthesis, 26 mm) due to Type A aortic dissection. Two months later, the patient presented with a rapidly enlarging false lumen of the remaining aortic arch and proximal descending aorta. A replacement of the distal aortic arch (one-branch Hemashield prosthesis; 30 mm) and descending aorta (Hemashield prosthesis, 22 mm) was performed under circulatory arrest via a left thoracotomy. The postoperative course was uneventful, and the patient was discharged on postoperative day 21.

**Table 1.** Patient admission date, indication, and treatment strategy.

Admission Date	Indication	Treatment
Dec 2009	Type A aortic dissection	• Full sternotomy, central cannulation, 26°C • Aortic root replacement with a biological valve conduit (Medtronic Freestyle-Aortic-Root Modell 995, Gr. 23 mm, Medtronic Inc, Minneapolis, MN) • Ascending aorta and partial arch replacement (Hemashield prosthesis, 26 mm) • Selective antegrade cerebral perfusion (sACP; 1000 mL/min)
Feb 2010	Rapidly enlarging aneurysma dissecans of the remaining aortic arch and proximal descending aorta	• Left lateral thoracotomy, femoro-femoral bypass, left ventricular venting, circulatory arrest at 25°C, antegrade cardioplegia administration using a Foley catheter (1800 mL Brettschneider solution), sACP (750 mL/min) • Replacement of distal aortic arch (one-branch Hemashield prosthesis, 30 mm) and descending aorta (Hemashield prosthesis, 22 mm)
June 2013	Aortoesophageal fistula ( Figure 1A )	• Full sternotomy, right axillary artery cannulation, sACP (750 mL/min), 25°C • Re-replacement of aortic arch and proximal descending aorta with a cryopreserved aortic homograft (CryoLife, Kennesaw, GA, 25 mm) • Closing of esophageal lesion with interrupted suture • Insertion of endoluminal esophageal stent (28/10 mm, Leufen-Medical-GmbH)
Oct 2013	Recurrent aortoesophageal fistula, new aortic rupture at the previous homograft site ( Figure 1B )	• Thoracic endovascular aortic repair stent over the aortic rupture (32/32/180, Valiant-Captivia, Medtronic, Santa Rosa, CA; Figure 2 ) • After stabilization: mediastinal debridement, esophageal resection, and gastric pull-up procedure with a cervical anastomosis (end-to-end, double-row suture with 3-0 Vicryl, Ethicon, Somerville, NJ)
Aug 2014	Infection of the stent graft ( Figure 3 )	• Patient refused aggressive treatment and was discharged on analgesia and broad-spectrum antibiotics for palliative care

**Figure 1.**
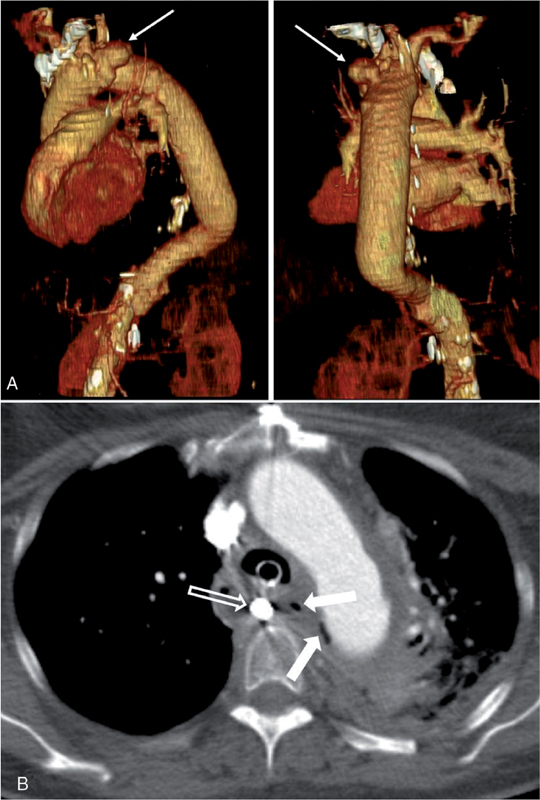
*Panel A* . A contained rupture between the distal aortic arch and descending aorta prosthesis (arrow) confirmed the presence of an aortoesophageal fistula. *Panel B* . Computed tomography (CT) scan showing the presence of air bubbles in the hematoma surrounding the thoracic aortic graft (arrow). A Segstaken-Blakemore tube was inserted into the esophagus (outlined arrow).

Three years later, the patient presented with recurrent hematemesis and anemia. Esophagogastroduodenoscopy (EGD) identified an esophageal ulcer with evidence of slight ongoing bleeding. Computed tomography (CT) examination confirmed the presence of an AEF ( Figure 1A ). An emergency re-replacement of the distal aortic arch and proximal descending aorta was performed with a cryopreserved aortic homograft (CryoLife, Kennesaw, GA; 25 mm). The adjacent esophageal lesion was closed during circulatory arrest with interrupted suture. An endoluminal esophageal stent (28/10 mm, Leufen-Medical-GmbH) was inserted at the end of the procedure. The stent was removed 2 months later, and control esophagogastroscopy demonstrated ulcerations with no visible fistula.

**Figure 2.**
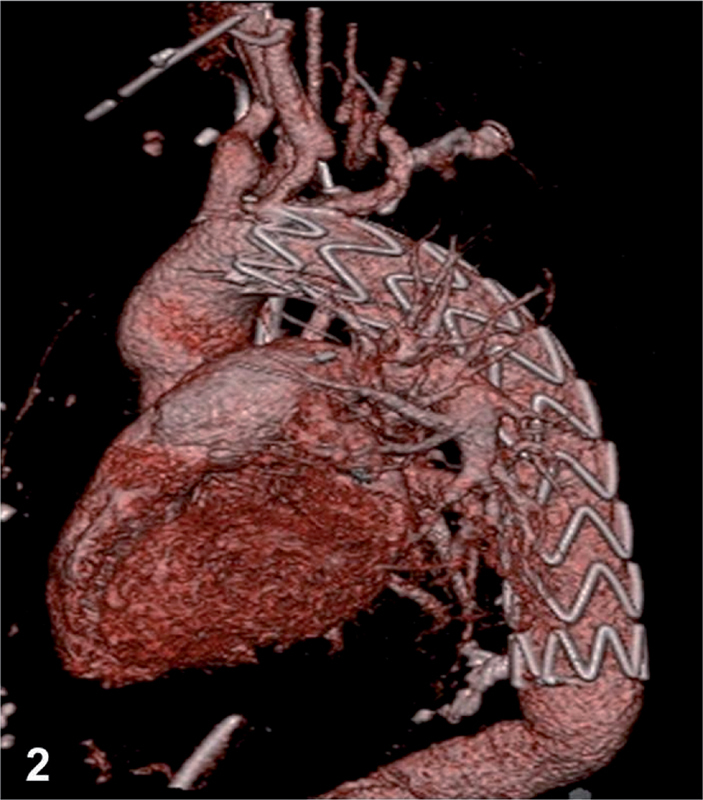
Three-dimensional reconstructed CT scan after thoracic endovascular stent graft implantation.

Six weeks post-discharge, the patient was readmitted due to massive hematemesis. Recurrent AEF was suspected, and the patient underwent emergent esophagogastroscopy. Active bleeding at the primary AEF location was observed, and a Segstaken-Blakemore tube was inserted. A CT scan was suspicious for a new aortic rupture at the previous homograft site ( Figure 1B ). An interdisciplinary team meeting resulted in the decision to place a TEVAR stent over the aortic rupture (32/32/180, Valiant-Captivia; Medtronic, Santa Rosa, Calif), which was successfully performed without incident ( Figure 2 ). After stabilization, the patient underwent mediastinal debridement, esophageal resection, and a gastric pull-up procedure with a cervical anastomosis (end-to-end, double-row suture with 3-0 Vicryl, Ethicon, Somerville, NJ). During the recovery phase, bronchoscopy revealed a 5-mm perforation of the trachea and a large amount of surrounding pus. Mediastinitis (secondary to *Enterococcus faecalis* and *Streptococcus anginosus* ) was diagnosed and treated with local debridement, vacuum-assisted closure therapy, and broad-spectrum antibiotics. The patient recovered well and was put on long-term antibiotics to prevent recurrent septicemia. A CT angiogram 5 months later confirmed satisfactory position of the implanted stent graft and showed no signs of endoleak or infection. Therefore, antibiotic therapy was discontinued.

**Figure 3.**
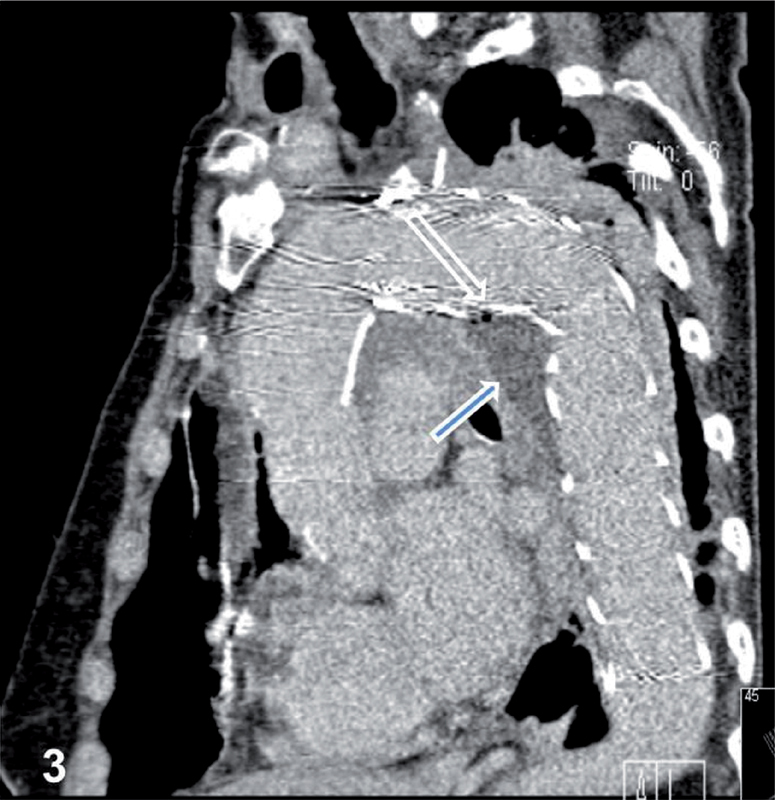
Computed tomography scan showing the stent graft surrounded by infected soft tissue (arrow) with air bubbles (outlined arrow) in the distal aortic arch and proximal descending thoracic aorta.

Ten months after the last admission, the patient experienced a persistent high-grade fever. A blood test revealed an elevated leukocyte count and highly elevated C-reactive protein level. CT revealed air around the stent grafts, suggesting infection ( Figure 3 ). After being informed that any further operative treatment (consisting of removal of the infected stent grafts and replacement of the descending aorta or extra-anatomic bypass grafting) was likely to prove fatal; the patient and her family decided to refuse the aggressive treatment. The woman was discharged on analgesia and broad-spectrum antibiotics for palliative care.

## Discussion

Patients who undergo surgery for aortic dissection often have residual dissected aortic tissue that may become a source of late complications. Repeat surgery is required in approximately 12–30% of patients, usually due to extension of dissection, aneurysm formation, or infection 1 . The very rapid progression of the distal arch and proximal descending aorta in our patient was caused by patent false lumen. CT scanning and intraoperative findings excluded infection of the prosthesis or presence of septic false aneurysm.

During follow-up, our patient developed an AEF at the site of the anastomosis between the original aortic arch replacement graft and the subsequent distal arch/descending aortic replacement graft. Secondary AEF following surgery is uncommon (4.8%), with 50% of cases occurring after aortic surgery 2 . The mechanism of AEF after conventional surgery involves rupture of the prosthesis, dehiscence of the repair, direct erosion of the graft into the esophagus, or local infection. Our intraoperative findings suggested that the cause of the AEF was dehiscence of the repair due to infection, which caused aortic rupture and secondary penetration into the esophagus. On the other hand, previous replacement of the descending aorta implicates the occlusion of esophageal arteries arising directly from the aorta with impaired tissue healing and possible esophageal ischemia 3 .

To treat the first AEF, we used a cryopreserved homograft to replace the distal aortic arch. Recent studies of AEF and aortoenteric fistulae demonstrate the superiority of cryopreserved aortic allografts because they are more resistant to infection 4 . However, homografts are not always immediately available in emergency situations. An alternative solution for orthotopic vascular reconstruction is use of self-made xenopericardial tube grafts constructed from a patch 5 . Recently, investigators from the European Registry of Endovascular Aortic Repair Complications presented the results of different treatment strategies for AEF following TEVAR at the 27th European Association for Cardio-Thoracic Surgery Annual Meeting in Vienna and concluded that radical esophagectomy and extensive aortic reconstruction is the only durable approach for this fatal complication.

When our patient re-presented with recurrent AEF, our interdisciplinary team decided against a fourth aortic arch operation because of the patient’s generally poor condition and the excessive operative risk. We therefore opted to perform TEVAR as a life-saving intervention, which was followed by esophageal resection and a gastric pull-up procedure in one stage with a cervical anastomosis and long-term antibiotic therapy. This treatment strategy resulted in immediate control of aortic bleeding and a complete regression of the recurrent AEF, but may have increased the subsequent risk of infection. Despite correction of the esophageal lesion, the efficacy of our therapy was limited by mediastinal infection, which required multiple surgical interventions. Prolonged postoperative antibiotic therapy is advocated as a key component for success, but there is currently no consensus on the appropriate duration of antibiotics in this group of patients 6 . Most commonly parenteral antibiotics are given for 2 to 8 weeks post-procedure, but whether lifelong oral antibiotics are necessary is debatable 6 . Most recently, Canaud et al. reviewed the outcomes of TEVAR for AEF and reported that prolonged antibiotic treatment (i.e., greater than 4 weeks) was associated with significantly lower aortic mortality 7 .

In our opinion, TEVAR for AEF can be used only as a bridge to definitive open aortic surgery or as combined treatment with mediastinal debridement, mediastinal drainage, and/or esophageal resection, particularly in patients in poor general condition. For long-term durability, it is necessary to resect the aorta and esophagus simultaneously to prevent prosthesis re-infection 8 . Based on our experience, stent graft infection can occur many months after the procedure. Thus, prolonged antibiotic therapy and life-long surveillance are mandatory in these patients regardless of symptoms or clinical signs of infection. However, additional clinical reports exclusively focusing on recurrent AEF are required to determine the optimal management strategy for this challenging problem.
